# Evolution of the ungulate dewlap: thermoregulation rather than sexual selection or predator deterrence?

**DOI:** 10.1186/s12983-016-0165-x

**Published:** 2016-07-18

**Authors:** Jakob Bro-Jørgensen

**Affiliations:** Mammalian Behaviour & Evolution Group, Department of Evolution, Ecology & Behaviour, Institute of Integrative Biology, University of Liverpool, Leahurst Campus, Neston, CH64 7TE UK

**Keywords:** Natural selection, Signal evolution, Ornaments, Male competition, Female Mate Choice, Claw-marks, Bovidae, Cervidae, Mammalia

## Abstract

**Background:**

Dewlaps are iconic features of several ungulate species and, although a role in signalling has been postulated, their function remains largely unexplored. We recently failed to find any age-independent link between dewlap size and social status in the common eland (*Tragelaphus oryx*), pointing to the possibility that sexual selection may not be the primary cause of dewlap evolution in ungulates. Here I use a two-pronged approach to test hypotheses on the function of ungulate dewlaps: an interspecific comparative analysis of bovids and deer, and an intraspecific study of eland antelopes in the wild.

**Results:**

Across species, the presence of dewlaps in males was not found to be associated with sexual size dimorphism, a commonly used measure of the intensity of sexual selection. The presence of dewlaps was, however, linked to very large male body size (>400 kg), which agrees with a thermoregulatory function as lower surface/volume-ratio counteracts heat dissipation in large-bodied species. In eland antelopes, large dewlap size was associated with higher, rather than lower, incidence of claw-marks (independently of age), a result which speaks against the dewlap as a predator deterrent and rather indicates a predation cost of the structure.

**Conclusion:**

The findings suggest that, although an additional function in communication should not be ruled out, the dewlap of ungulates may contrast with that of lizards and birds in thermoregulation being a primary function.

## Background

Extravagant ornament-like male morphologies that are sexually dimorphic are often assumed to have evolved by sexual selection to signal individual quality, either owing to female mate choice, male-male combat or both [1, 2]. However, often other explanations are possible and the importance of examining alternative hypotheses has recently been stressed [3]. Dewlaps, i.e. loose skin flaps hanging from the neck, are a case in point. These striking yet enigmatic structures are found in various vertebrate taxa, notably iguanid and agamid lizards, birds and ungulates. To date, studies of dewlap evolution and function have focused almost entirely on lizards and, to a lesser extent, birds. In lizards, a function of the often colourful dewlap in intraspecific communication is indicated by the fact that the dewlap is moveable and dewlap extensions constitute part of male territorial displays, with the display rate increasing during both intra- and intersexual encounters [4, 5]. However, recent studies suggest that rather than directly reflecting male competitive ability, a selective advantage of the dewlap may arise from drawing attention to head-bob and push-up displays [3]. In birds, dewlaps – often referred to as wattles – are present as a diverse set of fleshy excrescences pending from the neck in several taxa (in particular cassowaries and galliformes). Like in lizards, they are more pronounced in males than females and are thought to function as sexual signals of male quality [6]. The dewlap in ungulates has so far evaded rigorous investigation and its function remains a puzzle. In spite of some structural similarity with the dewlaps of lizards and birds, whether ungulate dewlaps have evolved as a result of the same selective pressures remains an open question.

**Fig. 1:**
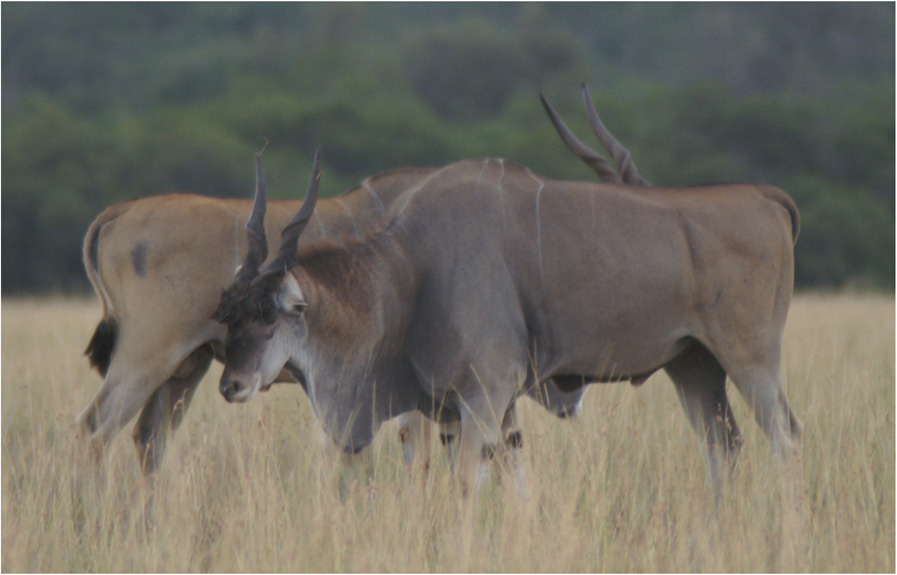
Broadside display between two eland bulls

As in lizards and birds, sexual dimorphism is pronounced in the dewlap of ungulates, but both sexual and natural selection could have affected the sexes differently to create this pattern. According to the ‘Sexual Selection Hypothesis’, the dewlap of ungulates, like in lizards and birds, has evolved because of a function in intra- and/or intersexual signalling. Different scenarios can be imagined. Firstly, the dewlap may be an honest signal of age-related fighting ability. Evidence from the common eland (*Tragelaphus oryx*) shows that dewlaps increase monotonically in size with age and the dewlap could thus provide meaningful information about fighting skills gained through experience [7, 8]. Another possibility is that the dewlap serves a deceptive function during rival assessment by exaggerating the body size perceived by opponents [3, 9]. Here it is worth noting that agonistic encounters in many ungulate species involve broadside displays where males assess the body size of rivals in lateral view; it is indeed from this perspective that the two-dimensional dewlap creates the most convincing illusion (Fig. 1).

Alternatively, according to the ‘Predator Deterrent Hypothesis’, the dewlap has its selective advantage in communication with predators rather than conspecifics [10]. Small ungulates are vulnerable to a wider range of predators than large ungulates [11, 12], and by enhancing apparent body size, dewlaps could thus deter predator attacks. Similar intimidating effects can explain a wide range of antipredator responses in vertebrates: piloerection in mammals, feather puffing in birds, anterior flattening in snakes, and body inflation in toads, frogs and fishes (although the latter also interferes with swallowing) [13]. Also conceivable is that the dewlap has evolved as a condition-dependent handicap signal [14]. According to this idea, the dewlap makes it easier for predators to get a hold on their prey, and because only ‘high quality’ individuals can develop large dewlaps without incurring prohibitive predation costs, the structure signals that an individual will be difficult to kill, thereby discouraging attacks. Such a condition-dependent handicap signal could also function as a ‘quality’ indicator that intimidates rivals and/or attracts mates, hence showing a potential link to the Sexual Selection Hypothesis.

Finally, according to the ‘Thermoregulation Hypothesis’, the selective advantage of the dewlap comes from facilitating the dissipation of excess body heat by convection, an idea proposed to explain the presence of dewlaps in dinosaurs [15, 16]. Overheating is a particular challenge for larger species owing to their lower surface/volume ratio, and the potential force of this selective pressure is illustrated by the evolution of large ears in elephants [17], where it is also noteworthy that the larger species has the proportionally largest ears (i.e. 6,300 kg of large males in the African elephant *Loxodonta africana* vs. 5,300 kg in the Asian elephant *Elaphus maximus*). Infrared measurements have confirmed the dewlap in the common eland as a site of high heat loss [18].

From these hypotheses follow various predictions at both the inter- and intraspecific level, and in this paper, I therefore combine a comparative approach, investigating differences between species, with an intraspecific field study on the common eland, a nomadic savannah antelope in which males develop large dewlaps drooping up to more than 40 cm beneath their necks. Quantifying predation is notoriously difficult in ungulate field studies because attacks usually take place when observation is difficult, i.e. at night and in dense vegetation. As a proxy measure, I therefore use claw-marks, the usefulness of which as an indicator of predation attempts have recently been highlighted [19]. Because the dewlap in ungulates is sexually dimorphic, the analyses are focused on males, where the structure is most pronounced.

According to the Sexual Selection Hypothesis, I predict that the presence of dewlaps among species is linked to sexual body size dimorphism, a frequently used index for the intensity of sexual selection [2, 3, 20]. According to the Predator Deterrent Hypothesis, where large dewlap size is associated with reduced attack rate by predators, I predict a negative correlation between dewlap size and the prevalence of claw-marks within species. Specifically, if dewlaps have evolved owing to the handicap principle, I predict that scars from past predation attempts will be located on the dewlap, by which the animal is hypothesized to be seized. According to the Thermoregulation Hypothesis, I predict that dewlaps are associated with large-bodied species, and that a threshold exists above which the low surface/volume ratio favours the evolution of dewlaps to facilitate heat dissipation.

## Methods

### Interspecific study: comparative analysis

A dataset was compiled focusing on all extant bovids and cervids including the following variables: presence/absence of dewlaps in males, mean male body mass, and sexual size dimorphism (SSD), which was calculated as male body mass divided by female body mass. The data were obtained from the sources: [9, 20–27]. The presence of dewlaps in males was entered as the binary response variable in a phylogenetic generalized linear mixed model for binary data (binary PGLMM). The analyses were performed in R [28] with the packages ‘ape’ [29, 30] and ‘caper’ [31]. The explanatory variables were male body mass and SSD (both log_10_-transformed); these were tested in bivariate models as well as in a multivariate model with backward elimination of non-significant predictors (*P* < 0.05). Control for evolutionary dependence was based on the phylogenetic tree reported in [32].

### Intraspecific study: eland field study

Study system: Eland males were studied between 2005 and 2013 in a 710 km^2^ area within the Masai Mara National Reserve and Olare Orok, Motorogi and Naboisho conservancies in Kenya. The habitat was dominated by open grass plains with only scattered thickets and hence good visibility. The eland is a large, sexually dimorphic antelope (males: 450–942 kg, females: 317–470 kg [33]), foraging on both grass and browse [34]. They are non-territorial and gregarious, their main social units being solitary males, male herds of 2–20 individuals, and larger mixed herds with up to more than a hundred individuals [8]. The main predator of male eland in the study area is the lion (*Panthera leo*); younger animals are occasionally killed by spotted hyenas (*Crocuta crocuta*), leopards (*Panthera pardus*) and, even more rarely, cheetahs (*Acinonyx jubatus*) [35–38].

**Fig. 2:**
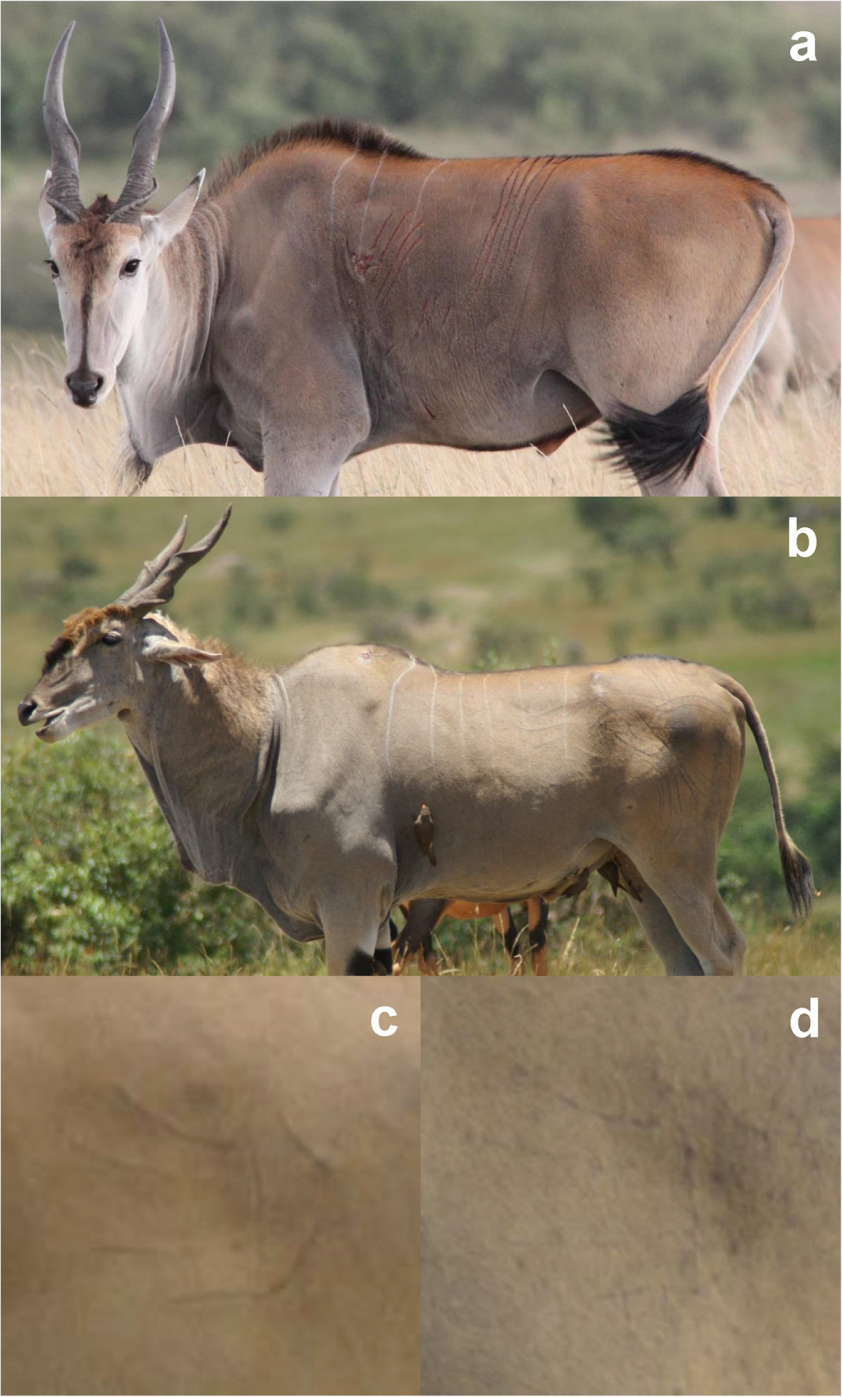
**a** A young male eland with claw-marks on his flank still bleeding. **b** An older male with claw-mark scars on his flank and hindquarters. **c** Claw-mark scars on the rump of a male estimated at 3 years old (2007). **d** The same scar as in **c** six years later (2013)

Data collection: As part of a larger study, data were collected annually between February and May during the wet season by surveying the study area for eland in a four-wheel-drive vehicle on a total of 266 days. For each observation, the identity of males were recorded based on their distinctive stripe pattern, ear nicks, scars, and tail and horn abnormalities, with identification details stored on paper sheets and in a digital library of bilateral photographs. For morphological measurements using photometry, individuals were photographed while standing relaxed, and their distance was measured by laser rangefinder (Bushnell Yardage pro 800). The measurements in pixels on the photographs were converted to the metric scale using a calibrated reference scale based on photographs of a 1 m pole at a range of distances [7]. Body size was measured as the body depth, i.e. the maximum vertical girth of the chest. Dewlap size was measured by the maximum vertical droop beneath the neck. Repeatability was high [7]. Scars from the claws of big cats, distinctive in the alignment of 4–5 scratches [19] (Fig. 2), were recorded as present/absent in the field, with supplementary observations in the digital photo library. Age was estimated upon first sighting of an individual, primarily based on horn rotation, horn wear, and development of hair fringes and hair tufts, following the protocol described in [8]. Complete measurements were obtained for 212 individual males.

Statistical analysis: The presence/absence of claw-marks was modelled as the response variable in two multivariate logistic regression models, a generalized linear mixed model (GzLMM) and a generalized linear model (GzLM). In the GzLMM, male identity was entered to control for correlated random effects owing to multiple measurements from the same individuals, whereas only one, randomly selected, observation per male was included in the GzLM. In both models, dewlap droop was included as a predictor variable, together with age and body size in order to control for confounding effects. Non-significant predictors were excluded by backward elimination (*P* < 0.05), and results relating to the non-significant predictors refer to their separate inclusion in the final model. Variance inflation factors (VIFs) were calculated to assess the potential for multicollinearity between dependent variables to affect the results [39]. These analyses were performed in SPSS version 21 (IBM, Armonk, NY, U.S.A.).

## Results

### Interspecific study: comparative analysis

Among species, the presence of dewlaps in males was predicted by large male body mass, but not by the degree of SSD (Table 1). These results agree with the Thermoregulation Hypothesis and speak against the Sexual Selection Hypothesis. All the species in which males had dewlaps were characterized by a mean male body mass above 400 kg, i.e. gaur (*Bos frontalis*, 848 kg), kouprey (*Bos sauveli*, approx. 800 kg), banteng (*Bos javanicus*, 750 kg), giant eland (*Tragelaphus derbianus*, 680 kg), common eland (647 kg), and moose (*Alces alces*, 440 kg); in less than 3 % of the species without dewlap did males weigh above this threshold (i.e. five out of 171 species; see Discussion).

**Table 1:** Predictors of the presence of dewlaps in males within the Bovidae and Cervidae (binary PGLMM: *N* = 133 species; phylogenetic signal: male body mass [log_10_]: s2 = 3.19, *P* < 0.001)

	Bivariate analyses			Multivariate analysis		
	Coefficient (±SEM)	*z*	*P*	Coefficient (±SEM)	*z*	*P*
Male body mass (log_10_)	3.59 ± 1.55	2.32	0.021	3.59 ± 1.55	2.32	0.021
Sexual body-size dimorphism (log_10_)	0.984 ± 2.538	0.39	0.698	-620 ± 6.32	-0.98	0.326

### Intraspecific study: eland field study

**Fig. 3:**
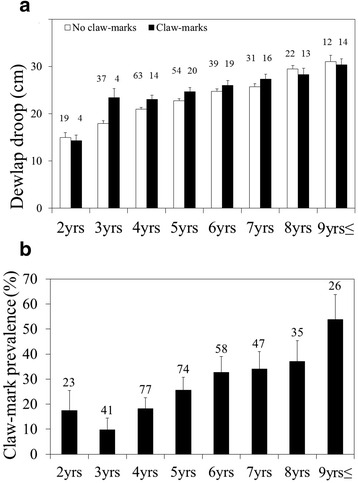
**a** Dewlap droop in males with and without claw-marks according to age. **b** Prevalence of claw-marks in relation to age. Columns and error bars indicate the mean and its standard error; numbers above columns refer to sample sizes

The incidence of claw-marks was positively, rather than negatively, related to dewlap size (logistic GzLMM: *N* = 381 observations on 212 males [34 of which were observed both with and without scars]; intercept: 3.67, *P* < 0.001; dewlap: t = 4.96, *P* < 0.001, VIF = 2.144; age: t = 1.553, *P* = 0.122, VIF = 2.596; body size: t = 0.385, *P* = 0.700, VIF = 2.118; logistic GzLM: *N* = 212 males: intercept: 3.05, *P* < 0.001; dewlap: : χ^2^ = 8.53, *P* = 0.004, VIF = 2.178; age: χ^2^ = 0.823, *P* = 0.364, VIF = 2.602; body size: χ^2^ = 0.028, *P* = 0.868, VIF = 2.113; Fig. 3a). This speaks against the Predator Deterrent Hypothesis and rather suggests a predation cost of large dewlaps. The robustness of the results to multicollinearity between dependent variables is supported by all VIFs being significantly below the threshold of 5. Contrary to the proposition that dewlaps may be condition-dependent handicaps, scars were not observed on the dewlap itself, with the majority of claw-marks located on the rump and flanks. The incidence of claw-marks increased from below 20 % at 2 years to above 50 % in animals of 9 years and above (Fig. 3b).

## Discussion

On the balance of the current evidence, benefits from thermoregulation emerge as a plausible main driver of the evolution of the ungulate dewlap (Table 2). Dewlaps have evolved independently among the very largest species within several ungulate radiations, namely the bovines, the tragelaphines and the cervids. Overheating is a particular problem for large species in hot climates, and the five species, in which males weigh above the 400 kg threshold but have not evolved dewlaps, either live in cold areas or have evolved alternative, behavioural, cooling mechanisms. The African buffalo (*Syncerus caffer*, 643 kg) and the water buffalo (*Bubalis bubalis*, 1,200 kg) regularly cool their body temperature by wallowing, and the wild yak (*Bos grunniens*, 591 kg), American bison (*Bison bison*, 795 kg) and European bison (*Bison bonasus*, 718 kg) inhabit boreal and temperate climates.

**Table 2:** Predictive framework and results

Predictions	Support?	Source
H1: Sexual selection hypothesis		
P1: SSD predicts the presence of dewlaps (interspecific)	No	This study
P2: Dewlap size predicts dominance status (intraspecific)	‘Yes’, but not after control for age	Bro-Jørgensen & Beeston (2015)
P3: Dewlap size predicts master bull status (intraspecific)	No	Bro-Jørgensen & Beeston (2015)
H2: Predator deterrence hypothesis		
P1: Large dewlap size predicts the absence of claw-marks (intraspecific)	No, the opposite	This study
P2: If condition-dependent handicap, scars from predation attempts present on dewlaps (intraspecific)	No	This study
H3: Thermoregulation hypothesis		
P1: Large body size predicts the presence of dewlaps (interspecific)	Yes (threshold approx. 400 kg)	This study

Neither the present interspecific study, nor our earlier results from the intraspecific study, provides support for the Sexual Selection Hypothesis. In male eland antelopes, we found no link between dewlap size and status as master bull in mixed-sex herds, and an association with dominance status in all-male herds disappeared after controlling for age [8]. Consistent with this, in the present study dewlaps were not found to have evolved more often in those species where pronounced sexual size dimorphism suggests intense sexual selection. While the sexual dimorphism of the dewlap itself, which is often minimal or absent in female ungulates, is consistent with the Sexual Selection Hypothesis, it is also consistent with the Thermoregulation Hypothesis as females are markedly smaller than males in all species with dewlaps; females are indeed close to the 400 kg threshold with weights around 350–450 kg in all species except the gaur (702 kg) and possibly the kouprey (no data).

At present, the most parsimonious interpretation of the link between claw-marks and large dewlap size is that benefits of large dewlaps in other contexts trade off against costs from increased predation risk, possibly because of lower running speed. However, conceivable is also an alternative explanation derived from the idea of the dewlap as a sexually selected handicap: rather than signalling the ability to flee, the dewlap could signal the ability to successfully fight off predators, in which case an association between large dewlaps and the presence of scars would indeed be expected. Still, against expectation remains the absence of scars on the dewlap itself since this is where the animals are hypothesized to be seized. At 30 %, the proportion of adult eland males with claw-marks, presumably primarily from lions, was comparable to figures found in another large savannah herbivore which share the lion as its main predator: the prevalence of claw-marks on adult female giraffes (*Giraffa camelopardalis*) in three study areas within the Serengeti-Mara ecosystem was found to be 4 %, 26 % and 32 % [19]. The prevalence of claw-marks may be related to the observation that eland do not usually flee from lions [33].

## Conclusions

All in all, the present evidence suggests that the dewlap in ungulates may be the result of convergent evolution under different selective pressures than those thought to underlie dewlaps in lizards and birds. Attaining very large body sizes in hot environments, the ungulate species in which dewlaps have evolved face a significant challenge to dissipate excess body heat. The structure is thus likely to facilitate temperature regulation, but a trade-off in terms of enhanced predation risk is suggested by the higher incidence of claw-marks in individuals with large dewlaps. Ruling out a communicative function of the dewlap on the basis of the present study would be premature and this remarkable trait, which has attracted considerable scientific attention in other taxa, warrants further study in ungulates.
